# Mitochondria-Targeting 1,5-Diazacyclooctane-Spacered Triterpene Rhodamine Conjugates Exhibit Cytotoxicity at Sub-Nanomolar Concentration against Breast Cancer Cells

**DOI:** 10.3390/ijms241310695

**Published:** 2023-06-27

**Authors:** Niels Heise, Selina Becker, Thomas Mueller, Matthias Bache, René Csuk, Antje Güttler

**Affiliations:** 1 Organic Chemistry, Martin-Luther-University Halle-Wittenberg, Kurt-Mothes-Str. 2, 06120 Halle (Saale), Germany; niels.heise@chemie.uni-halle.de (N.H.); selinabecker11@googlemail.com (S.B.); 2 University Clinic for Internal Medicine IV, Hematology/Oncology, Martin-Luther-University Halle-Wittenberg, Ernst-Grube-Str. 40, 06120 Halle (Saale), Germany; thomas.mueller@medizin.uni-halle.de; 3 Department of Radiotherapy, Martin-Luther-University Halle-Wittenberg, Ernst-Grube-Str. 40, 06120 Halle (Saale), Germany; matthias.bache@uk-halle.de (M.B.); antje.guettler@uk-halle.de (A.G.)

**Keywords:** asiatic acid, breast cancer, mitocans, rhodamine conjugates, triterpenoic acids

## Abstract

1,5-Diazacyclooctane was prepared by a simple synthetic sequence and coupled to pentacyclic triterpenoic acids oleanolic acid, ursolic acid, betulinic acid, platanic acid, and asiatic acid; these amides were activated with oxalyl chloride and reacted with rhodamine B or rhodamine 101 to yield conjugates. The conjugates were screened in SRB assays with various human breast cancer cell lines (MDA-MB-231, HS578T, MCF-7, and T47D) and found to exert cytotoxic activity even at a low concentration. Therefore, for an asiatic acid rhodamine 101 conjugate (28), an IC_50_ = 0.60 nM was determined and found to induce apoptosis in MDA-MB-231 and HS578T cells. Extra experiments showed the compound to act as a mitocan and to induce inhibition of proliferation or growth arrest in MDA-MB-231 cells at lower doses followed by an induction of apoptosis at higher doses. Furthermore, differential responses to proliferation inhibition and apoptosis induction may explain differential sensitivity of mammary cell lines to compound 28.

## 1. Introduction

Breast cancer is the most common type of tumor disease and, despite recent advances in cancer therapy, it remains the leading cause of tumor-related death in women [1,2,3,4,5,6,7,8]. While traditional treatments like surgery, chemotherapy, radiation, and hormone therapy are effective [9], they often cause severe side effects and may not be suitable for all patients. Therefore, there is a need to develop new and effective treatment options. One highly promising approach is the use of natural products derived compounds as anticancer agents, especially pentacyclic triterpenoids, which have emerged as a class of phytochemicals with potential anticancer activity. Several studies have demonstrated their ability to cause apoptosis, reduce clonogenic survival and migration, and enhance the radiosensitivity of human breast cancer cells [10,11,12,13]. These effects have been attributed to their ability to modulate various signaling pathways involved in cancer progression.

Pentacyclic triterpenoic acids linked with lipophilic cations, such as rhodamines [13,14,15,16,17,18,19,20,21,22,23,24,25,26,27], are known to act as mitocans even at low nanomolar concentrations by inhibiting their synthesis of ATP [21]. In this context, the mitochondrial targeting function of rhodamine seems particularly worth mentioning [28,29,30]. Therefore, the use of an amine spacer is crucial for enhancing their cytotoxicity, whereby secondary amines are favored over primary amines to prevent lactamization and maintain their cationic structures. Furthermore, incorporating a homopiperazinyl spacer leads to more cytotoxic compounds than those analogs with a piperazinyl spacer. Therefore, we have been interested in the use of a 1,5-diazacyclooctane spacer and its influence on the cytotoxicity of different pentacyclic triterpenoic acid conjugates of rhodamine B and rhodamine 101.

## 2. Results

**Scheme 1:**
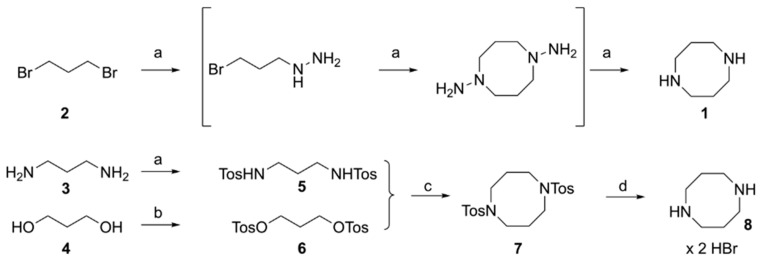
Synthesis of octahydro-1,5-diazocine (**1**) and its dihydrobromide (**8**): Reactions and conditions: a: NH_2_-NH_2_, EtOH, reflux, 4 h; then HBr, benzaldehyde, 7.5%; b: TosCl, no solvent, 80 °C, 30 min, 83%; c: TosCl, pyridine, 0 °C, 30 min, 87%; d: NaOMe, MeOH, DMF, 80 °C, 12 h, 84%; then HBr (33% glacial AcOH), 80 °C, 3 h, 92%.

Since the first preparation of octahydro-1,5-diazocine (**1,** 1,5-diazacyclooctane, a “bis-homo-piperazine”, Scheme 1) in 1939 by W. L. C. Veer [31] several routes have been suggested to this compound, among them the ring cleavage reaction of 1,5-diaminobicyclo [3.3.0]octane, the condensation of propane-1,3-diamine with 1,3-dibromopropane, and the silica-supported intramolecular cyclization of propane-1,3-diamine at 350 °C [32,33,34,35,36,37,38,39,40,41,42,43].

As an alternative, one could also imagine the reduction of the bis-lactam 1,5-diazocane-2,6-dione; the latter compound is accessible either via Staudinger ring closure reactions and Beckmann and Schmidt rearrangements, however, usually under very drastic conditions (e.g., fuming sulfuric acid) [44,45,46,47,48]. All these routes are not very suitable, since their mostly drastic conditions make the preparation of larger amounts on a laboratory scale quite difficult.

Special attention, therefore, is deserved for the only recently proposed [49] route starting from propane-1,3-diamine and propane-1,3-diol, two starting materials that are available in larger quantities and commercially cheap. In this process, both starting materials are first tosylated and then condensed by a double nucleophilic substitution. An alternative is the reaction of 1,3-dibromopropane (**2**) with hydrazine. This route would have the advantage of yielding the desired product in a one-pot procedure. However, it very quickly became apparent that many byproducts were formed in this reaction so that the maximum yield of pure **1** was 7.5% only. Working with larger quantities of hydrazine poses an additional risk.

However, the published synthesis using propane-1,3-diamine (**3**) and propane-1,3-diol (**4**) could not be reproduced in terms of the yields obtained either, so we decided to optimize this synthetic route on our own.

Thus, propane-1,3-diamine (**3**) was tosylated (Scheme 1) to yield **5** in an 83% yield, while the tosylation of propane-1,3-diol (**3**) gave 87% of the di-tosylate **6**. These two compounds were condensed in the presence of sodium methoxide (which proved to result in higher yields than using sodium ethoxide) to afford 84% of **7**. De-tosylation was performed with hydrobromic acid in the presence of thioanisole and the desired octahydro-1,5-diazocine was obtained as di-hydrobromide (**8**) in a 92% isolated yield.

**Figure 1:**
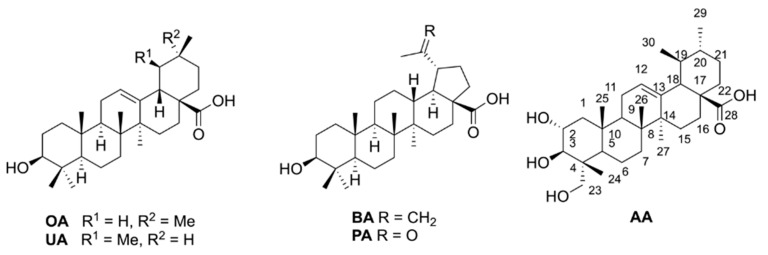
Structure of triterpenoic acid oleanolic acid (OA), ursolic acid (UA), betulinic acid (BA), platanic acid (PA), and asiatic acid (AA); for the latter, a numbering scheme is depicted.

The starting materials for the preparation of the spacered rhodamine conjugates were the triterpene carboxylic acids oleanolic acid (OA, Figure 1), ursolic acid (UA), and the lupanes betulinic acid (BA) and platanic acid (PA); in previous works, asiatic acid (AA) had been shown to be particularly suitable with respect to cytotoxic activity and was, therefore, included in this study as a model featuring a tri-hydroxylated triterpene carboxylic acid [21].

**Scheme 2:**
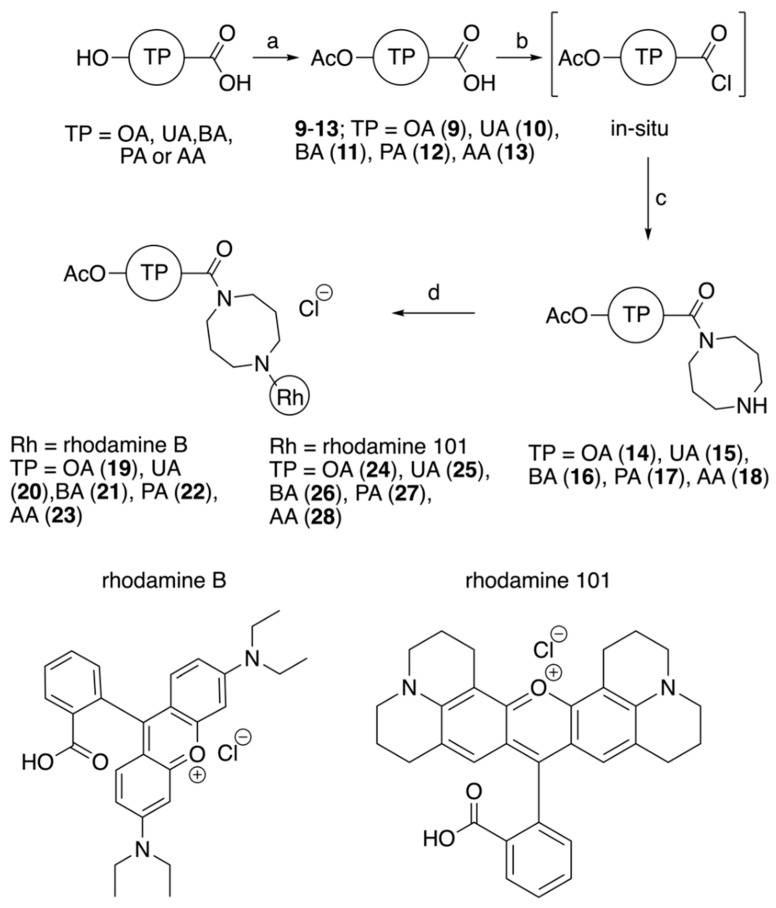
Synthesis of the rhodamine B and rhodamine 101 conjugates; reactions and conditions: a: Ac_2_O, DCM, NEt_3_, DMAP (cat.), 21 °C, 24 h; b: (COCl)_2_, DCM, DMF (cat.), in situ; c: DCM, **8**, NEt_3_, DMAP (cat.), 20 °C, 1 h; d: (COCl)_2_, DCM, DMF (cat.), then rhodamine B or rhodamine 101, 20 °C, 1 h.

The triterpenoic acids were acetylated to yield the acetates **9**–**13** (Scheme 2). Rhodamine B and rhodamine 101 were chosen as representative examples of rhodamines. The former compound has been shown in previous studies to be an essential component of mitocan-acting triterpene carboxylic acid amide conjugates; the latter differs from the former in having a somewhat higher lipophilicity (consensus log P_o/w_ 2.21 and 3.96, respectively; from www.swiss.adme.ch, accessed on 2 May 2023), which we consider advantageous for possible interactions with biological membranes. Thus, the reaction of acetates **9**–**13** with oxalyl chloride followed by the addition of **8** furnished amides **14**–**18**. Rhodamine B and rhodamine 101 were transformed with oxalyl chloride in situ into their corresponding acid chlorides that were reacted with amides **14**–**18** to yield rhodamine B-derived conjugates **19**–**23** and rhodamine 101-derived hybrids **24**–**28**.

Compounds **14**–**28** were screened in sulforhodamine B assays employing the breast cancer cell lines MDA-MB-231, HS578T, MCF-7, and T47D (Table 1). Breast cancer could be distinguished into different molecular subtypes: luminal-like (luminal A or B), HER2-enriched, and basal-like, which differ in biology, treatment response, patients’ survival, and clinical outcome. These subtypes are also found in cell lines and our investigated breast cancer cell lines have been characterized before. Breast cancer cell lines MDA-MB-231 and HS578T are basal and so-called triple negative, which means neither estrogen receptor (ER) and progesterone receptor (PR) nor human epidermal growth factor receptor 2 (HER2) are expressed. Basal breast cancers are mostly high-grade tumors and no therapeutic targeted therapy can be applied, thus resulting in a poor prognosis for patients although they are relatively sensitive for chemotherapy. MCF-7 and T47D breast cancer cells are luminal A and positive for ER and PR. Breast cancers of this type are often low-grade tumors, which are characterized by chemotherapy resistance, but hold good responses to hormone therapy, resulting in better clinical outcomes compared to basal breast cancers.

As a result, amides of triterpenoic acids 14–18 (Table 1) show cytotoxicity at a low micromolar range for all investigated breast cancer cell lines. IC_50_ values of about 0.5–50 µM were determined. As expected, conjugation of rhodamine B (compounds **19**–**23**) or rhodamine 101 (compounds **24**–**28**) led to increased cytotoxicity (in the nanomolar range) in all breast cancer cell lines (Table 1). In the investigated breast cancer cell lines, the IC_50_ values of all homopiperazinyl-spacered rhodamine B derivatives are in a low nano-molar range with rhodamine 101 conjugates being even more cytotoxic. An asiatic acid derivatized rhodamine 101 amide (compound **28**) is the most cytotoxic conjugate in all screened breast cancer cells. The IC_50_ values are in a low nanomolar range (0.6–126 nM). Comparing breast cancer cell lines, the HS578T cell line is the most resistant cell line for rhodamine B or rhodamine 101 conjugates (IC_50_ between 216 nM and 356 nM and between 126 nM and 1.3 µM). Our previous work showed that compounds of this class are also highly able to discriminate between malignant and nonmalignant cells [13,23] and affect mitochondrial ATP synthesis [23]. Future studies will also investigate whether changes in the expression of programmed death ligand-1 (PD-L1) can be observed [50].

In addition to studying the cytotoxicity of **28** in the above-mentioned cell lines, we investigated its ability to overcome resistance. While the IC_50_ of **28** in A2780 cells was 0.72 nM, the resistant A2780cis cells exhibited an IC_50_ of 1.82 nM. Although complete resistance reversal was not achieved, the results highlight the promising potential to partially overcome resistance. We also assessed its selectivity by comparing the cytotoxicity in nonmalignant fibroblasts CCD18Co. The IC_50_ value of **28** in CCD18Co cells was 503.2 nM, which was approximately 800-fold higher than the IC_50_ value observed in the MDA-MB-231 cells.

**Figure 2:**
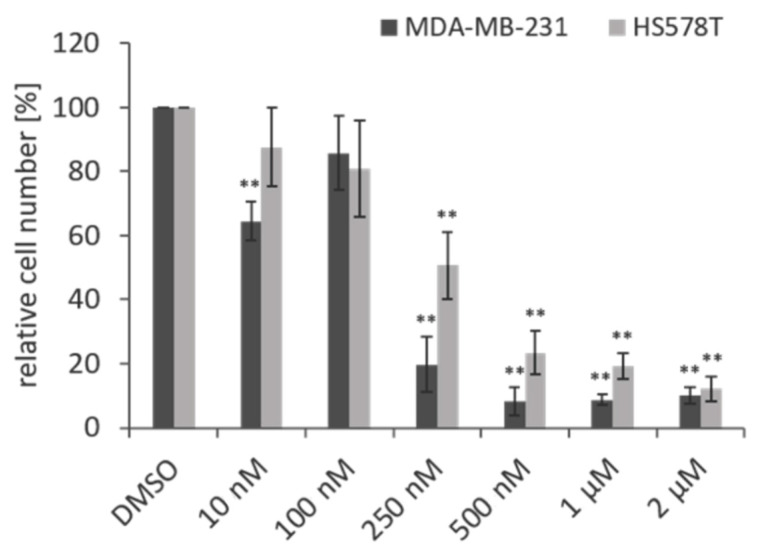
Relative cell number of MDA-MB-231 and HS578T breast cancer cells. Cells were seeded in 6-well plates and treated with different concentrations of compound 28. After 72 h the number of viable cells was counted. Data represent mean values (±SD) of at least three independent experiments. All data were referred to DMSO-treated cells (=100%). Significant *p* values are highlighted with asterisks (** *p* ≤ 0.01).

The most cytotoxic compound, **28**, was used for further investigations of proliferation and cell death in sensitive MDA-MB-231 and resistant HS578T breast cancer cells. In MDA-MB-231 cells, compound **28** caused a strong inhibition of proliferation (under 20% compared to the control cells) after treatment with at least 250 nM (Figure 2). However, in HS578T cells, treatment with 250 nM of compound **28** resulted in a less decrease of proliferation by about 50%, but with 500 nM, compound **28** cell number was reduced by up to 20% compared to control cells (Figure 2).

**Figure 3:**
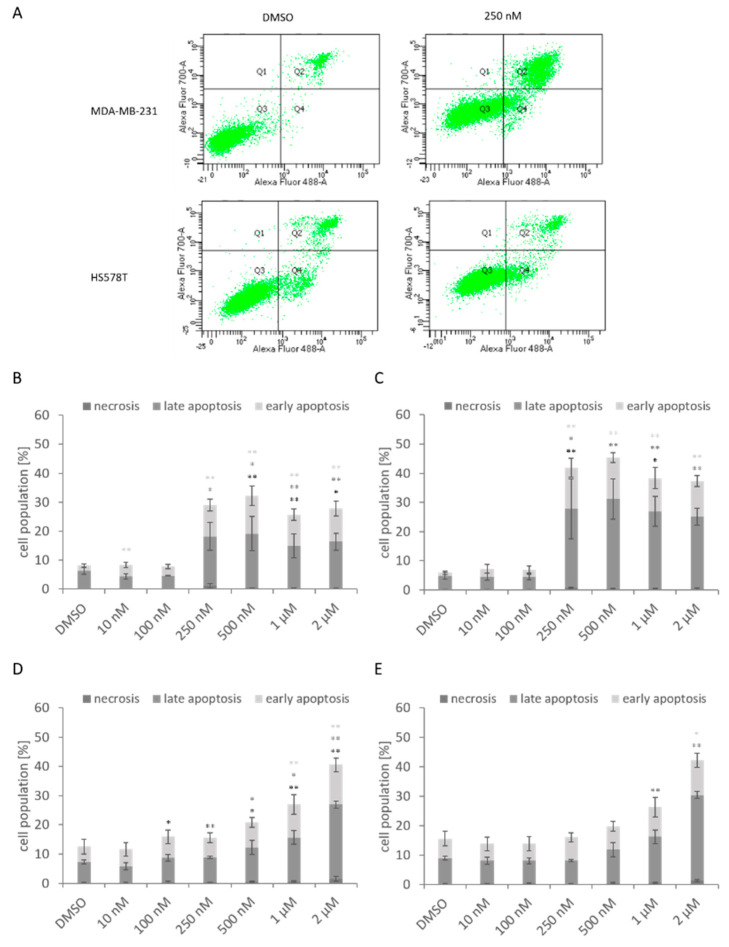
FITC Annexin V (Alexa 488)-Sytox Deep Red (Alexa 700) staining of MDA-MB-231 and HS578T cells. (**A**) Dot Plots of MDA-MB-231 and HS578T cell line after treatment with 250 nM compound 28 (**B**–**E**). Quantitative analysis of cell death of MDA-MB-231 (**B**,**C**) and HS578T cells (**D**,**E**) after treatment with different concentrations of compound 28 for 48 h (**B**,**D**) and 72 h (**C**,**E**). Data represent mean values (±SD) of at least three independent experiments. Significant *p* values are highlighted with asterisks (* *p* ≤ 0.05; ** *p* ≤ 0.01).

Cell death analyses were done by use of FITC annexin V-Sytox Deep Red staining in MDA-MB-231 (IC_50_ = 0.6 nM) and HS578T (IC_50_ = 126 nM) breast cancer cell lines to discriminate apoptotic and necrotic cells. An example of the evaluation of cell death via annexin V-Sytox Deep Red staining in the sensitive breast cancer cell line MDA-MB-231 and the resistant breast cancer cell line HS578T is shown in Figure 3A. Cells stained negative for both annexin V and Sytox Deep Red were viable (Q3). Early apoptotic cells stained positive for annexin V but negative for Sytox Deep Red (Q4), whereas late apoptotic or dead cells stained positive for both annexin V and Sytox Deep Red (Q2). Necrotic cells are indicated as negative for annexin V but positive for Sytox Deep Red (Q1).

**Figure 4:**
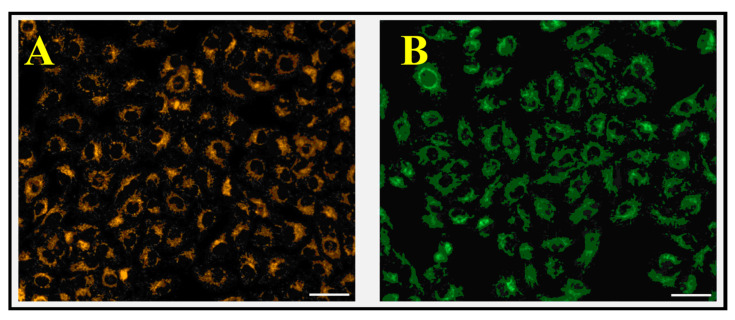
(**A**) Analysis of subcellular localization of compound **28** (**A**) was performed in MDA-MB-231 cells using BioTracker™ 488 Green Mitochondria Dye; (**B**) Cells treated with 100 nM 28 for 6 h or 100 nM BioTracker488 for 30 min, observed: BioTracker (475 nm/514 nm), AS101 (555 nm/592 nm). Scale bar: 50 µm.

Analysis of subcellular localization of compound **28** (Figure 4A) compared to the mitochondrial targeting compound BioTracker™ 488 Green Mitochondria Dye (Figure 4B) in MDA-MB-231 cells shows an identical pattern of accumulation, indicating the mitochondrial targeting of **28**. Using a quantitative analysis of the respective integrated fluorescence intensity, a mitochondrial uptake of about 56% could be determined.

In summary, the determination of proliferation and cell death indicates that compound **28** induces inhibition of proliferation or growth arrest at a lower dose, and with increasing dose treatment with compound **28** causes an induction of apoptosis. Furthermore, differential responses to proliferation inhibition and apoptosis induction may explain the differential sensitivity of mammary cell lines to compound **28**.

## 3. Discussion

1,5-Diazacyclooctane was synthesized through a straightforward synthetic pathway and subsequently linked with pentacyclic triterpenoic acids, namely oleanolic acid, ursolic acid, betulinic acid, platanic acid, and asiatic acid. These resulting amides were activated with oxalyl chloride and reacted with either rhodamine B or rhodamine 101 to form conjugates. These conjugates were then subjected to screening using SRB assays on various breast cancer cell lines, namely MDA-MB-231, HS578T, MCF-7, and T47D. The findings revealed that the conjugates exhibited cytotoxic activity even at low concentrations. Notably, the asiatic acid rhodamine 101 conjugate 28 displayed an IC_50_ = 0.60 nM and demonstrated the ability to induce apoptosis in MDA-MB-231 and HS578T cells. Further investigations demonstrated that the compound acted as a mitocan, resulting in the inhibition of proliferation or growth arrest in MDA-MB-231 cells at lower doses, followed by the induction of apoptosis at higher doses. Moreover, the differential responses observed in terms of proliferation inhibition and apoptosis induction could potentially explain the varying sensitivity of mammary cell lines to compound **28**.

## 4. Materials and Methods

### 4.1. General

NMR spectra were recorded using the Varian spectrometers (Darmstadt, Germany) DD2 and VNMRS (400 and 500 MHz, respectively). MS spectra were taken on an Advion expression^L^ CMS mass spectrometer (Ithaca, NY, USA; positive ion polarity mode, solvent: methanol, solvent flow: 0.2 mL/min, spray voltage: 5.17 kV, source voltage: 77 V, APCI corona discharge: 4.2 μA, capillary temperature: 250 °C, capillary voltage: 180 V, sheath gas: N_2_). Thin-layer chromatography was performed on precoated silica gel plates supplied by Macherey-Nagel (Düren, Germany). IR spectra were recorded on a Spectrum 1000 FT-IR-spectrometer from Perkin Elmer (Rodgau, Germany). The UV/Vis-spectra were recorded on a Lambda 14 spectrometer from Perkin Elmer (Rodgau, Germany); optical rotations were measured at 20 °C using a JASCO-P2000 instrument (JASCO Germany GmbH, Pfungstadt, Germany). The melting points (m.p.) were determined using the Leica hot-stage microscope Galen III (Leica Biosystems, Nussloch, Germany) and are uncorrected. The solvents were dried according to the usual procedures. Microanalyses were performed with an Elementar Vario EL (CHNS) instrument (Elementar Analysensysteme GmbH, Elementar-Straße 1, D-63505, Langenselbold, Germany).

All dry solvents were distilled over respective drying agents except for DMF which was distilled and stored under argon and a molecular sieve. Reactions using air- or moisture-sensitive reagents were carried out under an argon atmosphere in dried glassware. Triethylamine was stored over potassium hydroxide. Biological assays were performed as previously reported. The parent triterpenoic acids were obtained from local vendors.

### 4.2. General Procedure for Acetylation (GP 1)

To a solution of the parent triterpenoic acid (1 equiv.) in dry DCM, acetic anhydride (3 equiv.), dry triethylamine (3 equiv.), and DMAP (catal. amounts) were added, and the mixture was stirred at 20 °C for one day. The usual aqueous work-up followed by re-crystallization from ethanol furnished the corresponding acetates 9–13. Their respective m.p., ${\left[\alpha \right]}_{D}^{20}$ values, ^1^H, and ^13^C NMR spectra, as well as ESI MS data, correspond to the literature values.

### 4.3. General Procedure for the Synthesis of Amides **14**–**18** (GP 2)

To a solution of acetates 9–13 (1 equiv.) in dry DCM (100 mL), oxalyl chloride (5 equiv.) and DMF (2 drops) were added and the mixture was stirred at 20 °C for 2 h. The volatiles were removed under diminished pressure and the residue was dissolved in dry DCM (100 mL). This solution was slowly added to a solution of the corresponding amine (3 equiv.) in dry acetonitrile (100 mL) in the presence of DMAP (catal. amounts). The mixture was stirred at 20 °C for 1 day, the volatiles were removed under diminished pressure, and the residue was subjected to column chromatography (silica gel) to afford products **14**–**18**.

### 4.4. General Procedure for the Synthesis of the Rhodamine Conjugates **19**–**28** (GP 3)

To a solution of the rhodamine (rhodamine B or rhodamine 101, 1 equiv.) in dry DCM (100 mL), oxalyl chloride (7 equiv.) and dry DMF (2 drops) were added, and the mixture was stirred at 20 °C for 1 h. The volatiles were removed under diminished pressure and the residue was dissolved in dry DCM (100 mL). A solution of the corresponding amine (1 equiv.) in dry DCM (100 mL) was added, followed by the addition of catal. amounts of triethylamine and DMAP. The mixture was stirred at 20 °C for 1 h (TLC showed completion of the reaction), the solvents were removed in vacuo, and the residue was subjected to column chromatography (silica gel, CHCl_3_/MeOH) to afford products **19**–**28**.

### 4.5. N,N’-Ditosyl-1,3-propanediamine (**5**)

Tosyl chloride (40.0 g, 210 mmol) was molten in a beaker at 80 °C and 1,3-propanediamine (3, 8.9 mL, 106 mmol) was added dropwise; to complete the reaction, the mixture was stirred for an additional 30 min at 80 °C. After cooling to 20 °C, aq. HCl (2 m) was added, and the precipitate was washed with water followed by a recrystallization from ethanol to furnish **5** (33.7 g, 83%) as a colorless solid; m.p. 138 °C (lit: [49] 137–140 °C); R_f_ = 0.75 (silica gel, hexanes/ethyl acetate, 4:6); UV-Vis (CHCl_3_): λ_max_ (log ε) = 228 nm (4.16); IR (ATR): *ν* = 3271*w*, 1595*w*, 1431*w*, 1305*s*, 1214*w*, 1154*s*, 1088*m*, 1024*w*, 980*m*, 858*m*, 815*s*, 698*s*, 550*s*, 568*s*, 489*m* cm^−1^; ^1^H NMR (400 MHz, CDCl_3_): δ = 7.73 (*m*, 4H, 4-H, 8-H), 7.32–7.25 (*m*, 4H, 5-H, 7-H), 3.02 (*t*, *J* = 5.8 Hz, 4H, 2-H), 2.42 (*s*, 6H, 9-H), 1.67 (*p*, *J* = 6.2 Hz, 2H, 1-H) ppm; ^13^C NMR (101 MHz, CDCl_3_): δ = 143.6 (C-6), 136.8 (C-3), 129.8 (C-5, C-7), 127.0 (C-4, C-8), 39.8 (C-2), 29.9 (C-1), 21.5 (C-9) ppm; MS (ESI, MeOH/CHCl_3_, 4:1): *m*/*z* = 405.0 (100%, [M+Na]^+^).

### 4.6. 1,3-Propanediol Ditosylate (**6**)

A mixture of 1,3-propanediol (**4,** 16.0 g, 210 mmol) and tosyl chloride (88.0 g, 461 mmol) in dry pyridine (70 mL) was stirred at 0 °C for 1 h. The product was precipitated by adding aq. HCl (2 m), filtered off and dried. Compound **6** (69.9 g, 87%) was obtained as a colorless solid; m.p. 92 °C (lit.: [51] 92–93 °C); R_f_ = 0.49 (hexanes/ethyl acetate, 6:4); UV-Vis (CHCl_3_): λ_max_ (log ε) = 225 nm (4.11); IR (ATR): *ν* = 2978*w*, 1599*m*, 1496*w*, 1470*w*, 1421*w*, 1352*s*, 1293*m*, 1254*w*, 1190*m*, 1172*s*, 1095*m*, 1029*m*, 1021*m*, 941*s*, 892*w,* 852*s*, 810*s*, 739*s*, 660*s*, 580*s*, 568*s*, 549*s,* 488*m* cm^−1^; ^1^H NMR (400 MHz, CDCl_3_): δ = 7.75–7.23 (*m*, 8H, 4-H, 5-H, 7-H, 8-H), 4.06 (*t*, *J* = 6.0 Hz, 4H, 2-H), 2.46 (s, 6H, 9-H), 1.99 (*p*, *J =* 6.0 Hz, 2H, 1-H) ppm; ^13^C NMR (101 MHz, CDCl_3_): δ = 145.1 (C-6), 132.6 (C-3), 130.0 (C-5, C-7), 127.9 (C-4, C-8), 65.9 (C-2), 28.7 (C-1), 21.6 (C-9) ppm; MS (ESI, MeOH/CHCl_3_, 4:1): *m*/*z* = 407.3 (100%, [M+Na]^+^).

### 4.7. 1,5-Bis (p-Toluenesulfonyl)-1,5-diazacyclooctane (**7**)

To a solution of sodium methanolate (8.0 g, 148 mmol) in dry MeOH (100 mL) **5** (5.0 g, 13 mmol) was added, and the mixture was heated under reflux for 4 h. The solvent was removed, the residue was dissolved in dry DMF (100 mL) and 6 (5.0 g, 13 mmol) was added. The mixture was stirred at 80 °C for 12 h. The product was precipitated by adding aq. HCl (2 m), filtered off, and **7** (4.7 g, 84%) was obtained as a colorless solid; m.p. 214–216 °C (lit. [33]: 214–215 °C); R_f_ = 0.33 (hexane/ethyl acetate, 7:3); UV-Vis (CHCl_3_): λ_max_ (log ε) = 232 nm (4.32); IR (ATR): *ν* = 2953*w*, 1597*w*, 1456*m*, 1378*m*, 1321*s*, 1182*m*, 1150*s*, 1088*s*, 1017*m*, 1059*s*, 987*s*, 927*m*, 837*m*, 812*s*, 723*s,* 644*s*, 627*m*, 543*s*, 487*m*, 462*m*, 408*m* cm^−1^; ^1^H NMR (400 MHz, CDCl_3_): δ = 7.68 (*d*, *J* = 8.3 Hz, 4H, 5-H, 9-H), 7.33–7.30 (*m*, 4H, 6-H, 8-H), 3.31–3.24 (*m*, 8H, 1-H, 3-H), 2.43 (*s*, 6H, 10-H), 2.04 (*p*, *J* = 5.9 Hz, 4H, 2-H) ppm; ^13^C NMR (101 MHz, CDCl_3_): δ = 143.4 (C-7), 135.6 (C-4), 129.8 (C-6, C-8), 127.1 (C-5, C-9), 47.0 (C-1, C-3), 30.2 (C-2), 21.5 (C-10) ppm; MS (ESI, MeOH/CHCl_3_, 4:1): *m*/*z* = 445.2 (100%, [M+Na]^+^).

### 4.8. 1,5-Diazacyclooctane Dihydrobromide (**8**)

#### 4.8.1. Procedure A

A solution of **7** (2.5 g, 6 mmol) and thioanisole (2.4 mL, 18 mmol) in HBr (33% in glacial acetic acid, 150 mL) was stirred at 80 °C for 3 h. The volatiles were removed under diminished pressure, DCM (30 mL) was added, and the solution was washed with water (3 × 100 mL), followed by decolorization (activated charcoal). The solution was filtered, the solvent removed, and **8** (1.5 g, 5.5 mmol, 92%) was obtained as a colorless solid.

#### 4.8.2. Procedure B

A solution of hydrazine (75 mL, 1.5 mol) in EtOH (200 mL) was heated under reflux, and 1,3-dibromopropane (75 mL, 0.75 mol) was added slowly within 4 h. Stirring was continued for another hour, the solids were filtered off, washed with ethanol (3 × 50 mL), and discarded. The pH of the filtrate [combined with the EtOH washings and additional water (150 mL)] was adjusted to pH =3 by adding aqu. HBr (48% in water). Benzaldehyde (60 mL, 0.6 mol) was added, and the precipitate formed upon addition was filtered off, washed with water (3 × 50 mL), and discarded. The combined filtrates were extracted with ether (1000 mL), and the aq. The layer was concentrated under diminished pressure resulting in the formation of a red solid. Ethanol (250 mL) was added, and shaking of this suspension was continued for another 5 min. The yellowish solid was filtered off, washed with ethanol (250 mL) and ether (5 × 100 mL), and **8** (15.6 g, 7.5%) was obtained as a colorless solid; m.p. = 220–225 °C (lit.: [51,52] >250 °C); R_f_ = 0.8 (CHCl_3_:MeOH, 95:5); IR (ATR): *ν* = 2971*s*, 2728*s*, 2418*m*, 1577*s*, 1461*s*, 1331*m*, 1095*s*, 1027*m*, 890*m*, 696*m*, 547*m*, 491*m*, cm^−1^; ^1^H NMR (400 MHz, D_2_O): δ = 3.36–3.31 (*m*, 8H, 1-H, 3-H, 4-H, 6-H), 2.22–2.16 (*m*, 4H, 2-H, 5-H) ppm; ^13^C NMR (101 MHz, D_2_O): δ = 43.8 (C-1, C-3, C-4, C-6), 20.8 (C-2, C-5) ppm; MS (ESI, MeOH/CHCl_3_, 4:1): *m*/*z* = 115.0 (100%, [M+H-2 HBr]^+^).

### 4.9. (3ß)28-(1,5-Diazocan-1-yl)-28-oxoolean-12-en-3-yl Acetate (**14**)

Following GP 2 from 3-*O*-acetyl-oleanolic acid (9, 500 mg, 1.0 mmol), followed by chromatography (silica gel, CHCl_3_/MeOH (2% → 10%), compound **14** (425 mg, 71%) was obtained as a colorless solid; m.p. = 207–210 °C (decomp.); R_f_ = 0.52 (CHCl_3_/MeOH, 95:5); ${\left[\alpha \right]}_{D}^{20}$ = + 3.8° (*c* 0.088, CHCl_3_); IR (ATR): *ν* = 2954*m*, 1732*s*, 1626*m*, 1464*m*, 1368*s*, 1245*s*, 1026*s*, 750*s*, 662*w* cm^−1^; ^1^H NMR (400 MHz, CDCl_3_): δ = 5.25 (*m*, 1H, 12-H), 4.50 (*m*, 1H, 3-H), 3.67–3.11 (*m*, 8H, 33-H, 35-H, 36-H, 38-H), 3.03 (*d*, *J* = 13.8 Hz 1H, 18-H), 2.16–2.12 (m, 1H, 16-H), 2.04 (*s*, 3H, 32-H), 1.87–1.17 (*m*, 23H, 11-H, 34-H, 37-H, 19-H_a_, 2-H, 1-H_a_, 9-H, 6-H_a_, 15-H, 7-H, 21-H, 6 H_b_, 22-H, 19-H_b_), 1.13 (*s*, 3H, 27-H), 1.01–0.98 (m, 1H, 1-H_b_), 0.97-0.93 (*s*, 3H, 25-H), 0.92 (*s*, 3H, 30-H), 0.89 (*s*, 3H, 29-H), 0.85 (*s*, 3H, 23-H), 0.84 (*s*, 3H, 24-H), 0.82-0.81 (*m*, 1H, 5-H), 0.72 (*s*, 3H, 26-H) ppm; ^13^C NMR (101 MHz, CDCl_3_): δ = 177.0 (C-28), 171.2 (C-31), 144.7 (C-13), 121.7 (C-12), 81.1 (C-3), 55.5 (C-5), 47.9 (C-33, C-35, C-36, C-38), 47.8 (C-9),47.4 (C-17), 46.6 (C-19), 43.9 (C-18), 42.6 (C-14), 39.2 (C-8), 38.2 (C-1), 37.8 (C-4), 37.1 (C-10), 34.2 (C-21), 33.1 (C-7), 33.0 (C-29), 30.5 (C-34, C-37), 30.4 (C-20),29.8 (C-22), 28.2 (C-23), 27.6 (C-15), 26.0 (C-27), 24.1 (C-30), 23.7 (C-2), 23.5 (C-11), 22.8 (C-16), 21.4 (C-32), 18.3 (C-6), 17.3 (C-26), 16.8 (C-24), 15.6 (C-25) ppm; MS (ESI, MeOH/CHCl_3_, 4:1): *m*/*z* = 596.3 (100%, [M+H]^+^); analysis calcd. for C_38_H_62_N_2_O_3_ (594.93): C 76.72, H 10.50, N 4.71; found: C 76.47, H 10.74, N 4.50.

### 4.10. (3ß) 28-(1,5-Diazocan-1-yl)-28-oxours-12-en-3-yl Acetate (**15**)

Following GP 2 from 3-*O*-acetyl-ursolic acid (10, 500 mg, 1.0 mmol), followed by chromatography (silica gel, CHCl_3_/MeOH (2% → 10%), compound **15** (413 mg, 69%) was obtained as an off-white solid; m.p. = 232–235 °C (decomp.); R_f_ = 0.37 (CHCl_3_/MeOH, 95:5); ${\left[\alpha \right]}_{D}^{20}$ = + 0.45° (*c* 0.088, CHCl_3_); IR (ATR): *ν* = 2942*m*, 1731*m*, 1627*m*, 1456*m*, 1370*s*, 1245*s*, 1026*s*, 750*s*, 662*m* cm^−1^; ^1^H NMR (400 MHz, CDCl_3_): δ = 5.18–5.13 (*m*, 1H, 12-H), 4.46–4.39 (*m*, 1H, 3-H), 3.74–3.01 (*m*, 8H, 33-H, 35-H, 36-H, 38-H), 2.39 (*d*, *J* = 11.3 Hz, 1H, 18-H), 1.99 (*s*, 3H, 32-H), 1.88–1.82 (*m*, 2H, 11-H), 1.74–1.67 (*m*, 1H, 20-H), 1.73–1.05 (*m*, 23H, 2-H, 6-H, 15-H, 16-H, 21-H, 7-H, 9-H, 22-H, 1H_a_, 19-H, 34-H, 37-H), 1.02 (*s*, 3H, 27-H), 0.99–0.95 (m, 1H, 1H_b_), 0.92 (*s*, 3H, 23-H), 0.88 (*s*, 3H, 30-H), 0.86 (*s*, 3H, 25-H), 0.81 (*s*, 3H, 29-H), 0.80 (*s*, 3H, 24-H), 0.77-0.74 (*m*, 1H, 5-H), 0.72 (*s*, 3H, 26-H) ppm; ^13^C NMR (101 MHz, CDCl_3_): δ = 174.9 (C-28), 171.0 (C-31), 125.3 (C-12), 80.9 (C-3), 55.2 (C-5), 55.0 (C-18), 48.7 (C-33, C-35, C-36, C-38), 48. 6 (C-17), 47.7 (C-9), 43.4 (C- 8), 43.5 (C-14), 39.4 (C-19), 38.7 (C-20), 38.6 (C-1), 37.6 (C-4), 37.0 (C-10), 33.9 (C-22), 32.9 (C-7), 30.6 (C-21), 28.1 (C-23), 27.3 (C-34, C-37), 27.0 (C-15), 26.4 (C-16), 23,4 (C-27), 23.5 (C-2), 23.3 (C-11), 21.2 (C-32), 21.0 (C-30), 18.3 (C-6), 16.7 (C-29), 16.39 (C-26), 15.62 (C-24), 15.40 (C-25) ppm; MS (ESI, MeOH/CHCl_3_, 4:1): *m*/*z* = 596.2 (100%, [M+H]^+^); analysis calcd. for C_38_H_62_N_2_O_3_ (594.93): C 76.72, H 10.50, N 4.71; found: C 76.58, H 10.76, N 4.49.

### 4.11. (3ß) 28-(1,5-Diazocan-1-yl)-28-oxolup-20(29)-en-3-yl Acetate (**16**)

Following GP 2 from 3-*O*-acetyl-betulinic acid (11, 500 mg, 1.0 mmol), followed by chromatography (silica gel, ethyl acetate/MeOH (10% → 50%), compound **16** (430 mg, 72%) was obtained as a colorless solid; m.p. 223–234 °C (decomp.); R_f_ = 0.43 (CHCl_3_/MeOH, 9:1); ${\left[\alpha \right]}_{D}^{20}$ = −8.0° (*c* 0.064, CHCl_3_); IR (ATR): *ν* = 3408*w*, 2942*m*, 1731*m*, 1632*s*, 1455*m*, 1373*s*, 1246*s*, 1195*m*, 1026*m*, 979*m*, 882*m*, 730*s* cm^−1^; ^1^H NMR (400 MHz, CDCl_3_): δ = 4.68 (*m*, 1H, 29-H_a_), 4.56–4.53 (*m*, 1H, 29-H_b_), 4.42 (*dd*, J = 10.1, 6.2 Hz, 1H, 3-H), 4.02–3.19 (*m*, 8H, 33-H, 35-H, 36-H, 38-H), 2.85 (*m*, 2H, 13-H, 19-H), 2.13–2.08 (*m*, 1H, 16-H_a_), 2.00 (*s*, 3H, 32-H), 1.96–1.93 (*m*, 1H, 22-H_a_), 1.78–1.74 (*m*, 1H, 21-H_a_1.65–1.63 (*m*, 5H, 1-H_a_, 12-H_a_, 30-H), 1.60–1.05 (*m*, 2-H, 16-H_b_, 18-H, 6-H_a_, 7-H, 21-H, 11-H, 22-H_b_,34-H, 37-H, 9-H, 15-H), 0.96–0.94 (*m*, 1H, 1-H_b_), 0.92 (*s*, 3H, 27-H), 0.90–0.89 (*m*, 1H, 12-H_b_), 0.87 (*s*, 3H, 25-H), 0.80 (*s*, 3H,m 24-H), 0.79 (*s*, 3H, 23-H), 0.76–0.74 (*m*, 1H, 5-H), 0.72 (*s*, 3H, 26-H) ppm; ^13^C NMR (101 MHz, CDCl_3_): δ = 175.4 (C-28), 171.0 (C-31), 150.8 (C-20), 109.4 (C-29), 80.9 (C-3), 55.5 (C-5), 55.2 (C-33, C-35, C-36, C-38), 55.0 (C-17), 52.9 (C-18), 50.7 (C-9), 45.6 (C-19), 42.0 (C-14), 40.7 (C-8), 38.8 (C-4), 38.4 (C-1), 37.8 (C-10), 37.1 (C-7), 36.9 (C-13), 36.1 (C-22), 34.3 (C-34, C-37), 32.3 (C-16), 31.4 (C-21), 30.1 (C-15), 25.5 (C-23), 23.7 (C-12), 23.7 (C-2), 21.3 (C-32), 21.1 (C-11), 19.7 (C-30), 18.1 (C-6), 16.4 (C-24), 16.2 (C-25), 16.0 (C-26), 14.6 (C-27) ppm; MS (ESI, MeOH/CHCl_3_, 4:1): *m*/*z* = 596.1 (100%, [M+H]^+^); analysis calcd. for C_38_H_62_N_2_O_3_ (594.93): C 76.72, H 10.50, N 4.71; found: C 76.46, H 10.77, N 4.53.

### 4.12. (3ß)28-(1,5-Diazocan-1-yl)-30-nor-20,28-dioxolup-3-yl-acetate (**17**)

Following GP 2 from 3-*O*-acetyl-platanic acid (12, 500 mg, 1.0 mmol), followed by chromatography (silica gel, ethyl acetate/MeOH (10% → 50%), compound **17** (425 mg, 70%) was obtained as a colorless solid; m.p. = 210–214 °C (decomp.); R_f_ = 0.44 (CHCl_3_/MeOH, 9:1); ${\left[\alpha \right]}_{D}^{20}$ = −26.6° (*c* 0.028, CHCl_3_); IR (ATR): *ν* = 3396*w*, 2942*m*, 2866*m*, 1731*m*, 1626*s*, 1466*m*, 1411*m*, 1369*m*, 1197*s*, 1245*m*, 1120*m*, 1025*m*, 978*m* cm^−1^; ^1^H NMR (400 MHz, CDCl_3_): δ = 4.39 (*dd*, *J* = 10.6, 5.5 Hz, 1H, 3-H), 3.78–3.06 (*m*, 9H, 19-H, 32-H, 34-H, 35-H, 37-H), 2.66–2.56 (*m*, 1H, 13-H), 2.10 (*s*, 3H, 29-H), 2.08–1.98 (*m*, 2H, 16-H_a_, 18-H), 1.97 (*s*, 3H, 31-H), 1.94–1.90 (*m*, 1H, 22-H_a_), 1.82–1.76 (*m*, 1H, 21-H_a_), 1.70–1.05 (*m*, 19H, 1-H_a_, 16-H_b_, 2-H, 22-H_b_, 21-H_b_, 6-H_a_, 11-H_a_, 7-H, 6H_b_, 9-H, 15-H, 11-H_b,_ 33-H, 36-H), 0.98–0.95 (*m*, 2H, 12-H), 0.92 (*s*, 3H, 27-H), 0.91–0.85 (*m*, 1H, 1-H_b_), 0.83 (*s*, 3H, 24-H), 0.79–0.77 (*m*, 6H, 23-H, 25-H), 0.76 (*s*, 3H, 26-H), 0.72–0.71 (*m*, 1H, 5-H) ppm; ^13^C NMR (101 MHz, CDCl_3_): δ = 212.5 (C-20), 175.3 (C-28), 170.9 (C-30), 80.8 (C-3), 55.4 (C-5), 55.1 (C-32, C-34, C-35, C-37), 52.7 (C-18), 50.6 (C-9), 49.9 (C-19), 46.1 (C-17), 41.8 (C-8), 40.6 (C-14), 38.3 (C-1), 37.7 (C-4), 37.1 (C-10), 35.9 (C-13), 35.8 (C-22), 34.1 (C-7), 31.8 (C-16), 30.3 (C-29), 30.0 (C-15), 28.8 (C-21), 27.9 (C-23), 27.3 (C-12), 23.6 (C-2), 21.3 (C-31), 21.1 (C-11), 18.1 (C-6), 16.4 (C-26), 16.2 (C-25), 15.9 (C-24), 14.6 (C-27) ppm; MS (ESI, MeOH/CHCl_3_, 4:1): *m*/*z* = 597.3 (100%, [M+H^+^); analysis calcd. for C_37_H_60_N_2_O_4_ (596.90): C 74.45, H 10.13, N 4.69; found: C 74.21, H 10.32, N 4.43.

### 4.13. (2α,3ß,4α)28-(1,5-Diazocan-1-yl)-28-oxours-12-ene-2,3,23-triyl Triacetate (**18**)

Following GP 2 from 2,3,24-tri-*O*-acetyl-asiatic acid (13, 400 mg, 0.8 mmol), followed by chromatography [silica gel, CHCl_3_/MeOH (2% → 50%)], compound **18** (425 mg, 74%) was obtained as colorless solid; m.p. = 187–190 °C (decomp.); R_f_ = 0.38 (CHCl_3_/MeOH, 9:1); ${\left[\alpha \right]}_{D}^{20}$ = −30.2° (*c* 0.015, CHCl_3_); IR (ATR): *ν* = 2925*w,* 1741*s*, 1623*w*, 1368*m*, 1231*s*, 1042*m*, 748*w* cm^−1^; ^1^H NMR (400 MHz, CDCl_3_): δ = 5.22–5.17 (*m*, 1H, 12-H), 5.14–5.08 (*m*, 1H, 2-H), 5.04–5.01 (*m*, 1H, 3-H), 3.80 (*m*, 1H, 23-H_a_), 3.51 (*m*, 1H, 23-H_b_), 3.32–2.67 (*m*, 8H, 37-H, 39-H, 40-H, 42-H), 2.40–2.34 (*m*, 1H, 18-H), 2.03 (*s*, 3H, 36-H), 2.02–2.00 (*m*, 1H, 1-H_a_), 1.97 (*s*, 3H, 34-H), 1.92 (*s*, 3H, 32-H), 1.88–1.69 (*m*, 5H, 11-H, 16-H 22-H_a_), 1.60–1.45 (*m*, 4H, 22-H_b_, 9-H, 21-H_a_, 7-H_a_), 1.53–1.45 (*m*, 2H, 16-H_a_, 16-H_b_), 1.33–1.14 (*m*, 10H, 19-H, 5-H, 21-H_b_, 7-H_b_, 15-H, 38-H, 41-H), 1.11–1.09 (*m*, 1H, 1-H_b_), 1.05 (*s*, 3H, 27-H), 1.02 (s, 3H, 25-H), 0.99-0.96 (*m*, 1H, 20-H), 0.91 (*s*, 3H, 30-H), 0.84 (*s*, 3H, 24-H), 0.82 (*s*, 3H, 29-H), 0.70 (*s*, 3H, 26-H) ppm; ^13^C NMR (101 MHz, CDCl_3_): δ = 176.8 (C-28), 170.8 (C-35), 170.3 (C-33), 170.3 (C-31), 138.7 (C-13), 124.9 (C-12), 74.8 (C-3), 69.8 (C-2), 65.2 (C-23), 55.6 (C-18), 47.6 (C-9), 47.5 (C-5), 46.1 (C-37, C-39, C-40, C-42), 43.7 (C-1), 42.3 (C-14), 41.9 (C-4), 39.5 (C-8), 39.3 (C-19), 38.5 (C-20), 37.8 (C-10), 34.8 (C-22), 34.7 (C-7), 31.9 (C-21), 29.6 (C-15), 23.3 (C-11), 23.2 (C-16), 22.6 (C-38, C-41), 21.2 (C-30), 21.0 (C-36), 20.8 (C-32), 20.7 (C-34), 17.8 (C-6), 17.4 (C-27), 17.3 (C-29), 17.1 (C-25), 13.9 (C-26), 8.7 (C-24) ppm; MS (ESI, MeOH/CHCl_3_, 4:1): *m*/*z* = 711.8 (100%, [M+H^+^); analysis calcd. for C_42_H_66_N_2_O_7_ (711.00): C 70.95, H 9.36, N 3.94; found: C 70.69, H 9.51, N 3.75.

### 4.14. (3ß)-3-Acetyloxy-28-(5-{2-[3,6-bis(diethylamino)xanthen-10-ium-9-yl]benzoyl}-1,5-diazocan-1-yl)-28-oxoolean-12-ene Chloride (**19**)

Following GP 3 from **14** (150 mg, 0.14 mmol) and rhodamine B (100 mg, 0.2 mmol), followed by chromatography (silica gel, ethyl acetate/MeOH, 10% → 40%), **19** (100 mg, 72%) was obtained as a pink solid; m.p. = 211–216 °C; R_f_ = 0.44 (CHCl_3_/MeOH, 9:1); UV-Vis (CHCl_3_): λ_max_ (log ε) = 562 nm (4.53); IR (ATR): *ν* = 2926*m*, 2605*w*, 2498*w*, 1729*w*, 1587*s*, 1466*s*, 1412*s*, 1336, 1245*s*, 1180*s*, 1132*m*, 1073*s,* 1009*m*, 921*w*, 748*m*, 683*m* cm^−1^; ^1^H NMR (400 MHz, CDCl_3_): δ = 7.67–7.57 (*m*, 2H, 43-H, 44-H), 7.53–7.47 (*m*, 1H, 42-H), 7.34–7.26 (*m*, 3H, 45-H, 48-H), 7.14–6.65 (*m*, 4H, 49-H, 51-H), 5.25–5.15 (*m*, 1H, 12-H), 4.51–4.40 (*m*, 1H, 3-H), 3.78–3.20 (*m*, 16H, 33-H, 35-H, 36-H, 38-H, 53-H), 3.05–2.95 (*m*, 1H, 18-H), 2.07–2.02 (*m*, 1H, 16-H_a_), 2.01–1.99 (*m*, 3H, 32-H), 1.89–1.81 (*m*, 2H, 11-H), 1.67–1.37 (*m*, 14H, 19-H_a_, 1-H_a_, 2-H, 9-H, 6-H_a_, 15-H_a_, 22-H_a_, 21-H_a_, 6-H_b_, 34-H, 37-H), 1.30–1.24 (*m*, 12H, 54-H), 1.23–1.13 (*m*, 6H, 16-H_b_, 7-H 22-H_b_, 21-Hb, 19-H_b_,), 1.08 (*s*, 3H, 27-H), 0.99 (*m*, 2H, 1-H_b_, 15-H_b_), 0.87 (*s*, 3H, 25-H), 0.86 (*s*, 3H, 29-H), 0.84 (*s*, 3H, 30-H), 0.82 (*s*, 3H, 23-H), 0.80 (*s*, 3H, 24-H), 0.79–0.76 (*m*, 1H, 5-H), 0.68 (*s*, 3H, 26-H) ppm; ^13^C NMR (101 MHz, CDCl_3_): δ = 170.9 (C-28, C-31), 168.7 (C-39), 157.7 (C-52), 155.8 (C-46), 155.7 (C-50), 145.4 (C-13), 136.6 (C-41), 132.5 (C-49), 130.4 (C-40), 130.1 (C-42), 130.0 (C-44), 129.4 (C-43), 127.7 (C-45), 121.2 (C-129, 113.9 (C-47), 96.1 (C-48, C-51), 80.9 (C-3), 55.3 (C-5), 48.4 (C-17), 47.6 (C-9), 46.6 (C-19), 46.2 (C-53), 46.1 (C-33, C-35, C-36, C-38), 44.7 (C-18), 42.0 (C-14), 39.1 (C-8), 38.0 (C-1), 37.6 (C-4), 37.0 (C-10), 34.1 (C-21), 32.9 (C-30), 32.8 (C-22), 30.3 (C-20), 29.6 (C-7), 28.0 (C-15), 28.0 (C-23), 25.8 (C-27), 24.0 (C-29), 23.5 (C-2), 23.3 (C-11), 22.6 (C-16), 21.3 (C-32), 18.2 (C-6), 17.2 (C-26), 16.6 (C-24), 15.4 (C-25), 12.7 (C-54) ppm; MS (ESI, MeOH/CHCl_3_, 4:1): *m*/*z* = 1021.4 (98%, [M-Cl]^+^); analysis calcd. for C_66_H_91_N_4_O_5_Cl (1055.93): C 75.07, H 8.69, N 5.31; found: C 74.87, H 8.82, N 5.08.

### 4.15. (3ß)-3-Acetyloxy-28-(5-{2-[3,6-bis(diethylamino)xanthen-10-ium-9-yl]benzoyl}-1,5-diazocan-1-yl)-28-oxours-12-ene Chloride (**20**)

Following GP 3 from **15** (150 mg, 0.14 mmol) and rhodamine B (100 mg, 0.2 mmol), followed by chromatography (silica gel, ethyl acetate/MeOH, 10% → 40%), **20** (94 mg, 63%) was obtained as a pink solid; m.p. = 194–197 °C (decomp.); R_f_ = 0.41 (CHCl_3_/MeOH, 9:1); UV-Vis (CHCl_3_): λ_max_ (log ε) = 560 nm (5.54); IR (ATR): *ν* = 2932*w*, 1726*w*, 1586*s*, 1465*m*, 1411*s*, 1335*s*, 1272*m*, 1245*s*, 1179*s*, 1132*m*, 1073*m*, 1009*m*, 921*m,* 823*w*, 746*m*, 683*m*, 663*m*, 498*m* cm^−1^; ^1^H NMR (400 MHz, CDCl_3_): δ = 7.68–7.56 (*m*, 2H, 43-H, 44-H), 7.53–7.49 (*m*, 1H, 42-H), 7.32–7.25 (*m*, 3H, 45-H, 48-H), 7.18–6.54 (*m*, 4H, 49-H, 51-H), 5.24–5.11 (*m*, 1H, 12-H), 4.50–4.39 (*m*, 1H, 3-H), 4.12–2.76 (*m*, 16H, 33-H, 35-H, 36-H, 38-H,53-H), 2.43–2.33 (*m*, 1H, 18-H), 2.09–2.07 (*m*, 1H, 16-H_a_), 2.00 (*s*, 3H, 32-H), 1.91–1.84 (*m*, 2H, 11-H), 1.77–1.36 (*m*, 14H, 1-H_a_, 2-H, 21-H_a_, 6-H_a_, 9-H, 22-H_a_, 19-H, 6-H_b_, 16-H_b_, 34-H, 37-H), 1.29 (*t*, J = 7.1 Hz, 12H, 54-H), 1.23 (*m*, 6H, 7-H, 15-H, 21-H_b_, 22-H_b_), 1.03 (s, 4H, 1-H_b_, 27-H), 0.96–0.93 (*m*, 1H, 20-H), 0.90 (*s*, 3H, 29-H), 0.88 (*s*, 3H, 25-H), 0.83 (*s*, 3H, 30-H), 0.82 (*s*, 3H, 23-H), 0.81 (*s*, 3H, 24-H), 0.77–0.75 (*m*, 1H, 5-H), 0.69 (*s*, 3H, 26-H) ppm; ^13^C NMR (101 MHz, CDCl_3_): δ = 170.9 (C-28), 157.7 (C-31), 157.7 (C-39), 155.8 (C-50), 155.7 (C-46) 155.6 (C-52), 136.6 (C-40), 132.9 (C-48), 130.1 (C-44), 130.0 (C-42), 129.4 (C-43), 127.0 (C-45), 125.0 (C-12), 113.9 (C-47), 96.2 (C-49, C-51), 80.9 (C-3), 55.3 (C-18), 55.3 (C-5), 49.4 (C-17), 47.5 (C-9), 46.2 (C-33, C-35, C-36, C-38), 46.1 (C-53), 42.4 (C-14), 39.6 (C-19), 39.3 (C-8), 38.6 (C-20), 38.2 (C-1), 37.6 (C-4), 36.9 (C-10), 32.7 (C-22), 31.9 (C-7), 30.5 (C-21), 29.6 (C-15), 29.3 (C-16), 28.0 (C-23), 23.2 (C-2), 23.1 (C-11), 23.0 (C-27), 18.1 (C-6), 17.4 (C-30), 17.2 (C-26), 16.7 (C-24), 15.5 (C-25), 12.7 (C-54) ppm; MS (ESI, MeOH/CHCl_3_, 4:1): *m*/*z* = 1020.4 (100%, [M-Cl]^+^); analysis calcd. For C_66_H_91_N_4_O_5_Cl (1055.93): C 75.07, H 8.69, N 5.31; found: C 74.83, H 8.91, N 5.03.

### 4.16. (3ß)-3-Acetyloxy-28-(5-{2-[3,6-bis(diethylamino)xanthen-10-ium-9-yl]benzoyl}-1,5-diazocan-1-yl)-28-oxolup-20(29)-ene Chloride (**21**)

Following GP 3 from **16** (300 mg, 0.5 mmol) and rhodamine B (200 mg, 0.4 mmol), followed by chromatography (silica gel, CHCl_3_/MeOH, 9:1), **21** (3536 mg, 69%) was obtained as a pink solid; m.p. = 212–218 °C; R_f_ = 0.49 (CHCl_3_/MeOH, 9:1); UV-Vis (CHCl_3_): λ_max_ (log ε) = 562 nm (4.43); IR (ATR): *ν* = 2936*w*, 1730*w*, 1587*s*, 1465*m*, 1411*s*, 1335*s*, 1244*s*, 1179*s*, 1132*m*, 1073*m*, 978*w,* 921*w*, 684*m* cm^−1^; ^1^H NMR (400 MHz, CDCl_3_): δ = 7.52–7.47 (*m*, 2H, 43-H, 44-H), 7.43–7.37 (*m*, 1H, 42-H), 7.22–6.99 (*m*, 3H, 45-H, 48-H), 6.97–6.71 (*m*, 2H, 49-H), 6.64–6.57 (*m*, 2H, 51-H), 4.58–4.50 (*m*, 1H, 29-H_a_), 4.42–4.37 (*m*, 1H, 29-H_b_), 4.30 (*m*, 1H, 3-H), 3.73–2.93 (*m*, 16H, 33-H, 35-H, 36-H, 38-H, 53-H), 2.83–2.68 (*m*, 2H, 19-H, 13-H), 2.00–1.94 (*m*, 1H, 16-H_a_), 1.87 (*s*, 3H, 32-H), 1.83–1.54 (*m*, 4H, 22-H_a_, 15-H, 21-H_a_), 1.53–1.51 (*m*, 2H, 12-H_a_, 1-H_a_), 1.50 (*s*, 3H, 30-H), 1.47–1.42 (*m*, 2H, 2-H), 1.41–1.36 (*m*, 1H, 18-H), 1.35–1.29 (*m*, 2H, 16-H_a_, 6-H_a_), 1.28–1.12 (*m*, 21H, 11-H_a_, 6-H_b_, 7-H, 22-H_b_, 54-H, 34-H, 37-H), 1.11–1.09 (*m*, 2H, 11-H_b_, 9-H, 21-H_b_), 0.83–0.74 (*m*, 8H, 1-H_b_, 12-H_b_, 23-H, 27-H), 0.69–0.65 (*m*, 9H, 26-H, 25-H, 24-H), 0.63–0.59 (*m*, 1H, 5-H) ppm; ^13^C NMR (101 MHz, CDCl_3_): δ = 170.8 (C-28), 168.5 (C-39), 167.6 (C-31), 157.6 (C-50), 155.6 (C-46), 155.5 (C-52), 151.2 (C-20), 136.4 (C-40), 132.3 (C-41), 130.8 (C-48), 130.0 (C-43), 129.4 (C-42), 128.6 (C-45), 127.0 (C-44), 114.6 (C-49), 113.6 (C-47), 108.9 (C-29), 96.0 (C-51), 80.8 (C-3), 55.2 (C-5), 55.1 (C-38, C-36, C-35, C-33), 53.0 (C-18), 50.6 (C-9), 46.1 (C-53), 45.8 (C-19), 41.9 (C-17), 40.6 (C-8), 40.6 (C-14), 38.3 (C-1), 37.7 (C-10), 37.0 (C-4), 36.8 (C-13), 36.0 (C-22), 34.2 (C-7), 32.0 (C-16), 31.3 (C-21), 30.2 (C-34, C-37), 29.8 (C-15), 27.8 (C-24), 25.5 (C-12), 25.4 (C-37, C-34), 23.6 (C-2), 21.2 (C-32), 21.0 (C-11), 19.6 (C-30), 18.1 (C-6), 16.1 (C-25), 15.9 (C-26), 14.6 (C-23), 14.5 (C-27), 12.6 (C-54) ppm; MS (ESI, MeOH/CHCl_3_, 4:1): *m*/*z* = 1020.5 (100%, [M-Cl]^+^); analysis calcd. for C_66_H_91_N_4_O_5_Cl (1055.93): C 75.07, H 8.69, N 5.31; found: C 74.86, H 8.90, N 5.09.

### 4.17. (3ß)-3-Acetyloxy-28-(5-{2-[3,6-bis(diethylamino)xanthen-10-ium-9-yl]benzoyl}-1,5-diazocan-1-yl)-28-oxolup-20-oxo Chloride (**22**)

Following GP 3 from **17** (300 mg, 0.50 mmol) and rhodamine B (300 mg, 0.6 mmol), followed by chromatography (silica gel, ethyl acetate/MeOH, 9:1), **22** (350 mg, 69%) was obtained as a pink solid; m.p. = 198–201 °C; R_f_ = 0.51 (CHCl_3_/MeOH, 9:1); UV-Vis (CHCl_3_): λ_max_ (log ε) = 558 nm (4.73); IR (ATR): *ν* = 2934*w*, 1721*m*, 1585*s*, 1410*m*, 1466*s*, 1334*s*, 1272*s*, 1245*s*, 1131*s*, 1072*s*, 1009*s*, 977*m*, 921*m*, 823*m*, 755*m*, 682*s* cm^−1^; ^1^H NMR (400 MHz, CDCl_3_): δ = 7.67–7.57 (*m*, 2H, 42-H, 43-H), 7.53–7.47 (*m*, 1H, 41-H), 7.35–7.27 (*m*, 3H, 44-H, 47-H), 7.11–6.64 (*m*, 4H, 48-H, 50-H), 4.46–4.36 (*m*, 1H, 3-H), 3.88–3.20 (*m*, 16H, 32-H, 34-H, 35-H, 37-H, 52-H), 3.16–3.05 (*m*, 1H, 18-H), 2.76–2.51 (*m*, 1H, 13-H), 2.11–2.04 (*m*, 4H, 16-H_a_, 29-H), 1.98 (*s*, 4H, 19-H, 31-H), 1.78 (*s*, 2H, 21-H_a_, 22-H_a_), 1.62–1.48 (*m*, 4H, 1-H_a_, 2-H, 16-H_a_), 1.42 (*m*, 7H, 6-H_a_, 22-H_b_, 21-H_b_, 11-H, 7-H_a_, 6-H_b_), 1.28 (*t*, *J* = 6.8 Hz, 14H, 7-H_b_, 9-H, 53-H), 1.23–1.04 (*m*, 6H, 33-H, 36-H, 15-H), 0.94 (*s*, 2H, 12-H), 0.91 (*s*, 4H, 1-H_b_, 24-H), 0.82 (*s*, 3H, 27-H), 0.78 (*m*, 10H, 23-H, 25-H, 26-H, 5-H) ppm; ^13^C NMR (101 MHz, CDCl_3_): δ = 212.8 (C-20), 170.9 (C-28, C-30, C- 38), 157.7 (C-49), 155.7 (C-45), 155.6 (C-51), 136.6 (C-39), 136.5 (C-40), 132.4 (C-47), 130.1 (C-42), 129.7 (C-43), 129.4 (C-44), 127.1 (C-41), 114.5 (C-48), 113.7 (C-46), 96.2 (C-50), 80.8 (C-3), 55.4 (C-5), 55.3 (C-32, C-34, C-35, C-37), 53.0 (C-19), 50.6 (C-9), 50.3 (C-18), 49.5 (C-17), 46.2 (C-52), 41.9 (C-14), 40.6 (C-8), 38.3 (C-1), 37.7 (C-4), 37.1 (C-10), 35.8 (C-13), 35.6 (C-22), 34.2 (C-7), 31.6 (C-16), 30.1 (C-29), 29.9 (C-15), 28.8 (C-21), 27.9 (C-23), 27.4 (C-12), 23.6 (C-2), 22.6 (C-33, C-36), 21.1 (C-11), 18.1 (C-6), 16.4 (C-25), 16.2 (C-26), 14.7 (C-24), 14.0 (C-27), 12.7 (C-53) ppm; MS (ESI, MeOH/CHCl_3_, 4:1): *m*/*z* = 1022.4 (100%, [M-Cl]^+^); analysis calcd. for C_65_H_89_N_4_O_6_Cl (1057.90): C 73.80, H 8.48, N 5.30; found: C 73.55, H 8.67, N 5.07.

### 4.18. (2α,3ß,4α)2,3,23-Tris (acetyloxy)-28-(5-{2-[3,6-bis(diethylamino)xanthen-10-ium-9-yl]benzoyl}-1,5-diazocan-1-yl)-28-oxours-12-en Chloride (**23**)

Following GP 3 from **18** (300 mg, 0.4 mmol) and rhodamine B (250 mg, 0.5 mmol), followed by chromatography (silica gel, ethyl acetate/MeOH, 9:1), **23** (184 mg, 60%) was obtained as a pink solid; m.p. = 225 °C; R_f_ = 0.44 (CHCl_3_/MeOH, 9:1); UV-Vis (CHCl_3_): λ_max_ (log ε) = 562 nm (4.50); IR (ATR): *ν* = 2927*w*, 1793*m*, 1587*s*, 1467*m*, 1411*m*, 1336*s*, 1244*s*, 1179*s*, 1042*m*, 921*w*, 684*m*, 436*w* cm^−1^; ^1^H NMR (400 MHz, CDCl_3_): δ = 7.66–7.57 (*m*, 2H, 47-H, 48-H), 7.52–7.47 (*m*, 1H, 46-H), 7.27-7.20 (m, 49-H, 52-H), 7.17–6.63 (*m*, 4H, 53-H, 55-H), 5.20–5.00 (*m*, 3H, 12-H, 2-H, 3-H), 3.82–3.76 (*m*, 1H, 23-H_a_), 3.73–2.93 (*m*, 17H, 37-H, 39-H, 40-H, 42-H, 57-H, 23-H_b_), 2.44–2.33 (*m*, 1H, 18-H), 2.04 (*s*, 3H, 34-H), 2.02–1.99 (*m*, 1H, 1-H_a_), 1.97 (*s*, 3H, 36-H), 1.93 (*s*, 3H, 32-H), 1.90–1.32 (*m*, 15H, 11-H, 9-H, 15-H, 16-Ha, 21-H_a_, 22-H_a_, 20-H, 38-H, 41-H, 6-H), 1.28 (*t*, *J* = 7.1 Hz, 13H, 5-H, 58-H), 1.25–1.10 (*m*, 5H, 7-H, 16-H_b_, 21-H_b_, 22-H_b_), 1.09–1.07 (*m*, 1H, 1-H_b_), 1.04 (*s*, 3H, 30-H), 1.01 (*s*, 3H, 27-H), 0.97–0.92 (*m*, 1H, 19-H), 0.88 (*s*, 3H, 29-H), 0.84 (*s*, 3H, 25-H), 0.81 (*s*, 3H, 26-H), 0.69 (*s*, 3H, 24-H) ppm; ^13^C NMR (101 MHz, CDCl_3_): δ = 176.2 (C-43), 170.8 (C-35), 170.4 (C-31, C-33), 170.3 (C-28), 157.7 (C-54), 155.7 (C-56), 155.6 (C-50), 138.6 (C-13), 136.6 (C-45), 132.3 (C-52), 130.1 (C-47), 130.0 (C-49), 129.3 (C-48), 127.2 (C-46), 125.9 (C-12), 113.9 (C-51), 96.2 (C-53, C-55), 74.8 (C-3), 69.9 (C-2), 65.3 (C-23), 55.5 (C-18), 53.4 (C-37, C-39, C-40, C-42), 47.6 (C-5), 47.5 (C-9), 46.2 (C-57), 46.1 (C-17), 43.7 (C-1), 42.5 (C-4), 41.9 (C-14), 38.9 (C-8), 38.7 (C-20), 38.6 (C-19), 37.8 (C-10), 32.6 (C-22), 30.5 (C-21), 29.6 (C-7), 28.4 (C-15), 23.4 (C-27), 23.3 (C-11), 21.2 (C-29), 21.0 (C-32), 20.8 (C-36), 20.7 (C-34), 17.8 (C-6), 17.4 (C-26), 17.2 (C-30), 17.0 (C-24), 13.9 (C-25), 12.6 (C-58) ppm; MS (ESI, MeOH/CHCl_3_, 4:1): *m*/*z* = 1036.5 (100%, [M-Cl]^+^); analysis calcd. for C_70_H_95_N_4_O_9_Cl (1172.00): C 71.74, H 8.17, N 4.78; found: C 71.49, H 8.35, N 4.47.

### 4.19. 3β-Acetyloxy-28-[4-[3-(2,3,6,7,12,13,16,17-octahydro-1H,5H,11H,15H-pyrido [3,2,1-ij]pyrido[1’’,2’’,3’’:1’,8’]quinolino[6’,5’:5,6]pyrano[2,3-f ]quinolin-4-ium-9-yl)benzoyl]1,5-diazocan-1-yl]-28-oxo-olean-12-en Chloride (**24**)

Following GP 3 from **14** (100 mg, 0.14 mmol) and rhodamine 101 (200 mg, 0.4 mmol), followed by chromatography (silica gel, ethyl acetate/MeOH, 10% → 50%), **24** (114 mg, 75%) was obtained as a pink solid; m.p. = 205–210 °C; R_f_ = 0.41 (CHCl_3_/MeOH, 9:1); UV-Vis (CHCl_3_): λ_max_ (log ε) = 580 nm (4.23); IR (ATR): *ν* = 2942*w,* 1727*m*, 1595*s*, 1493*m*, 1459*m*, 1362*m*, 1295*s*, 1196*s*, 1035*m*, 746*m,* 420*m* cm^−1^; ^1^H NMR (400 MHz, CDCl_3_): δ = 7.81–7.61 (*m*, 2H, 45-H), 7.54–7.43 (*m*, 1H, 42-H), 7.29 (*s*, 1H, 44-H), 6.86–6.47 (*m*, 2H, 48-H), 5.27–5.21 (*m*, 1H, 12-H), 4.51–4.44 (*m*, 1H, 3-H), 3.79–3.15 (*m*, 16H, 33-H, 35-H, 36-H, 38-H, 52-H, 57-H), 3.09–2.90 (*m*, 5H, 18-H, 55-H,), 2.76–2.47 (*m*, 4H, 50-H), 2.15–2.06 (*m*, 4H, 56-H), 2.03 (*s*, 3H, 32-H), 1.97 (*s*, 5H, 16-H_a_, 51-H), 1.85 (*s*, 2H, 11-H), 1.70–1.15 (*m*, 20H, 19-H_a_, 21-H, 2-H, 1-H_a_, 9-H, 6-H_a_, 7-H_a_, 6-H_b_, 22-H_a_, 7-H_b_, 15-H, 22-H_b_, 19-H_b_, 34-H, 37-H), 1.12 (*s*, 3H, 30-H), 1.07–0.97 (*m*, 2H, 1-H_b_, 16-H_b_), 0.91 (*s*, 3H, 25-H), 0.90 (*s*, 3H, 27-H), 0.88 (*s*, 3H, 29-H), 0.85 (*s*, 3H, 23-H), 0.83 (*s*, 3H, 24-H), 0.81–0.79 (*m*, 1H, 5-H), 0.72 (*s*, 3H, 26-H) ppm; ^13^C NMR (101 MHz, CDCl_3_): δ = 171.0 (C-28, C-31, C-39), 164.1 (C-53), 152.0 (C-46), 139.9 (C-58), 134.6 (C-41), 131.0 (C-40), 130.5 (C-44), 129.4 (C-45, C-42), 126.9 (C-43), 125.9 (C-48), 123.5 (C-47), 121.4 (C-12), 113.0 (C-49), 105.3 (C-54), 81.0 (C-3), 55.4 (C-5), 51.1 (C-33, C-35, C-36, C-38), 50.5 (C-52, C-57), 48.2 (C-17), 47.6 (C-9), 46.6 (C-19), 43.7 (C-18), 43.3 (C-14), 39.1 (C-8), 38.1 (C-1), 37.7 (C-4), 37.0 (C-10), 33.9 (C-22), 33.0 (C-29), 32.9 (C-7), 30.5 (C-20), 30.3 (C-21), 29.7 (C-15), 28.0 (C-23), 27.8 (C-16), 27.6 (C-50), 25.8 (C-30), 24.1 (C-27), 23.5 (C-2), 23.4 (C-11), 21.3 (C-32), 20.6 (C-51), 19.9 (C-55), 19.7 (C-56), 18.2 (C-6), 17.2 (C-26), 16.7 (C-24), 15.4 (C-25) ppm; MS (ESI, MeOH/CHCl_3_, 4:1): *m*/*z* = 1068.6 (100%, [M-Cl]^+^); analysis calcd. For C_70_H_91_N_4_O_5_Cl (1103.97): C 76.16, H 8.31, N 5.08; found: C 75.81, H 8.52, N 4.89.

### 4.20. 3β-Acetyloxy-28-[4-[3-(2,3,6,7,12,13,16,17-octahydro-1H,5H,11H,15H-pyrido[3,2,1-ij]pyrido[1’’,2’’,3’’:1’,8’]quinolino[6’,5’:5,6]pyrano[2,3-f]uinoline-4-ium-9-yl)benzoyl]1,5-diazocan-1-yl]-28-oxo-urs-12-en Chloride (**25**)

Following GP 3 from **15** (150 mg, 0.2 mmol) and rhodamine 101 (150 mg, 0.3 mmol), followed by chromatography (silica gel, ethyl acetate/MeOH, 10% → 50%), **25** (94 mg, 62%) was obtained as a pink solid; m.p. = 199–202 °C; R_f_ = 0.43 (CHCl_3_:Methanol, 9:1); UV-Vis (CHCl_3_): λ_max_ (log ε) = 571 nm (3.94); IR (ATR): *ν* = 3388*w,* 2925*m*, 1728*m*, 1597*s*, 1495*m*, 1459*m*, 1362*s*, 1297*s*, 1246*s*, 1195*s*, 1100*s*, 1024*s,* 421*s* cm^−1^; ^1^H NMR (400 MHz, CDCl_3_): δ = 8.36–8.06 (*m*, 1H, 43-H), 7.75–7.63 (*m*, 2H, 42-H, 45-H), 7.24–7.11 (*m*, 1H, 44-H), 6.81–6.50 (*m*, 2H, 48-H), 5.26–5.16 (*m*, 1H, 12-H), 4.49–4.43 (*m*, 1H, 3-H), 3.71–3.21 (*m*, 16H, 33-H, 35-H, 36-H, 38-H, 52-H, 57-H), 3.17–2.90 (*m*, 4H, 55-H), 2.81–2.57 (*m*, 4H, 50-H), 2.23–2.06 (*m*, 4H, 56-H), 2.01 (*s*, 3H, 32-H), 1.98–1.84 (*m*, 6H, 51-H, 11-H_a_, 16-H_a_), 1.66–1.19 (*m*, 22H, 1-H_a_, 11-H_b_, 21-H_a_, 6-H_a_, 22-H_a_, 19-H, 6-H_b_, 21-H_b_, 22-H_b_, 2-H, 15-H, 7-H, 16-H_b_, 18-H, 34-H, 37-H), 1.13–1.10 (*m*, 3H, 29-H), 1.05 (*s*, 3H, 27-H), 1.04–0.99 (*m*, 2H, 1-H_b_, 20-H), 0.92 (*s*, 3H, 24-H), 0.85 (*s*, 3H, 25-H), 0.83 (*s*, 3H, 23-H), 0.82 (*s*, 3H, 30-H), 0.79–0.77 (*m*, 1H, 5-H), 0.72 (*s*, 3H, 26-H) ppm; ^13^C NMR (101 MHz, CDCl_3_): δ = 171.0 (C-28), 169.3 (C-31, C-39), 151.2 (C-53), 150.9 (C-46), 135.2 (C-58), 132.3 (C-42), 131.3 (C-43), 130.2 (C-45), 129.1 (C-44), 127.1 (C-48), 125.1 (C-12), 112.6 (C-49), 111.6 (C-47), 105.6 (C-54), 80.9 (C-3), 55.3 (C-5), 47.7 (C-33, C-35, C-36, C-38), 47.5 (C-18), 47.5 (C-9), 45.3 (C-17), 43.3 (C-52, C-57), 41.7 (C-14), 39.6 (C-8), 39.5 (C-19), 38.6 (C-20), 38.2 (C-1), 37.7 (C-4), 36.9 (C-10), 33.1 (C-22), 31.9 (C-7), 30.5 (C-21), 29.7 (C-15), 28.0 (C-16), 27.8 (C-23), 27.5 (C-50), 25.0 (C-34, C-37), 23.5 (C-11), 23.4 (C-27), 23.3 (C-51), 22.6 (C-2), 21.3 (C-32), 19.9 (C-55), 19.7 (C-56), 18.7 (C-29), 18.1 (C-6), 17.3 (C-30), 16.7 (C-26), 15.5 (C-24), 14.1 (C-25) ppm; MS (ESI, MeOH/CHCl_3_, 4:1): *m*/*z* = 1068.4 (100%, [M-Cl]^+^); analysis calcd. for C_70_H_91_N_4_O_5_Cl (1103.97): C 76.16, H 8.31, N 5.08; found: C 75.87, H 8.59, N 4.83.

### 4.21. 3β-Acetyloxy-28-[4-[3-(2,3,6,7,12,13,16,17-octahydro-1H,5H,11H,15H-pyrido[3,2,1-ij] pyrido[1”,2”,3”:1’,8’]quinolino[6’,5’:5,6]pyrano[2,3-f]quinolin-4-ium-9-yl)benzoyl] 1,5-diazocan-1-yl]-28-oxo-lup-20(29)-en Chloride (**26**)

Following GP 3 from **16** (200 mg, 0.14 mmol) and rhodamine 101 (200 mg, 0.4 mmol), followed by chromatography (silica gel, CHCl_3_/MeOH, 9:1), **26** (103 mg, 68%) was obtained as a pink solid; m.p. = 203–206 °C; R_f_ = 0.44 (CHCl_3_/MeOH, 9:1); UV-Vis (CHCl_3_): λ_max_ (log ε) = 578 nm (4.33); IR (ATR): *ν* = 2931*w,* 1721*w*, 1595*s*, 1493*s*, 1361*m*, 1294*s*, 1246*s*, 1180*s*, 1035*s*, 746*m*, 622*m*, 421*s* cm^−1^; ^1^H NMR (400 MHz, CDCl_3_): δ = 7.68–7.53 (*m*, 2H, 43-H, 45-H), 7.52–7.45 (*m*, 1H, 42-H), 7.31–7.26 (*m*, 1H, 44-H), 6.76–6.59 (*m*, 2H, 48-H), 4.70–4.62 (*m*, 1H, 29-H_a_), 4.54–4.49 (*m*, 1H, 29-H_b_), 4.44–4.38 (*m*, 1H, 3-H), 3.81–3.02 (*m*, 16H, 33-H, 35-H, 36-H, 38-H, 52-H, 57-H), 3.00–2.89 (*m*, 4H, 55-H), 2.88–2.72 (*m*, 2H, 13-H, 18-H), 2.71–2.51 (*m*, 4H, 50-H), 2.16–2.01 (*m*, 5H, 16-H_a_, 56-H), 1.99 (*s*, 3H, 32-H), 1.96–1.87 (*m*, 5H, 21-H_a_, 51-H), 1.85–1.74 (*m*, 2H, 15-H_a_, 22-H_a_), 1.70–1.66 (*m*, 1H, 12-H_a_), 1.62 (*s*, 4H, 1-H_a_, 30-H), 1.60–1.53 (*m*, 2H, 2-H), 1.50–1.47 (*m*, 1H, 9-H), 1.47–1.40 (*m*, 2H, 6-H_a_, 16-H_b_), 1.36–1.06 (*m*, 13H, 11-H_a_, 21-H_b_, 6-H_b_, 7-H, 15-H_b_, 19-H, 22-H_b_, 11-H_b_, 34-H, 37-H), 0.94 (*s*, 2H, 1-H_b_, 12-H_b_), 0.90 (*s*, 3H, 24-H), 0.86 (*s*, 3H, 25-H), 0.81 (*s*, 3H, 27-H), 0.79 (*s*, 3H, 23-H), 0.73 (*s*, 3H, 26-H), 0.70–0.59 (*m*, 1H, 5-H) ppm; ^13^C NMR (101 MHz, CDCl_3_): δ = 170.9 (C-28), 168.8 (C-31, C-39), 151.9 (C-53), 151.3 (C-46), 151.2 (C-20), 138.4 (C-40, C-41), 136.5 (C-58), 130.4 (C-44), 129.4 (C-45, C-42), 127.1 (C-48), 127.0 (C-43) 123.5 (C-47), 113.1 (C-49), 109.1 (C-29), 105.3 (C-54), 81.0 (C-3), 55.5 (C-5), 53.1 (C-9), 52.9, 50.9 (C-33, C-35, C-36, C-38), 50.7 (C-19), 50.5 (C-52, C-57), 49.4 (C-17), 45.9 (C-13), 42.0 (C-14), 40.7 (C-14), 40.6 (C-8), 38.4 (C-1), 37.8 (C-4), 37.1 (C-10), 36.9 (C-18), 36.1 (C-21), 34.3 (C-7), 32.1 (C-16), 31.4 (C-22), 29.9 (C-15), 27.9 (C-23), 27.5 (C-50), 25.5 (C-12), 23.7 (C-2), 22.6 (C-34, C-37), 21.3 (C-32), 21.1 (C-11), 20.6 (C-51), 19.8 (C-55), 19.6 (C-56), 18.7 (C-30), 18.2 (C-6), 16.5 (C-25), 16.4 (C-26), 14.7 (C-24), 14.6 (C-27) ppm; MS (ESI, MeOH/CHCl3, 4:1): *m*/*z* = 1067.2 (100%, [M-Cl]^+^); analysis calcd. for C_70_H_91_N_4_O_5_Cl (1103.97): C 76.16, H 8.31, N 5.08; found: C 75.98, H 8.52, N 4.83.

### 4.22. 3β-Acetyloxy-28-[4-[3-(2,3,6,7,12,13,16,17-octahydro-1H,5H,11H,15H-pyrido[3,2,1-ij] pyrido[1”,2”,3”:1’,8’]quinolino[6’,5’:5,6]pyrano[2,3-f]quinolin-4-ium-9-yl)benzoyl] 1,5-diazocan-1-yl]-30-nor-20,28-dioxo-lup-20(29)-en Chloride (**27**)

Following GP 3 from **17** (200 mg, 0.3 mmol) and rhodamine 101 (100 mg, 0.2 mmol), followed by chromatography (silica gel, CHCl_3_/MeOH, 9:1), **27** (132 mg, 60%) was obtained as a pink solid; m.p. = 208–210 °C; R_f_ = 0.49 (CHCl_3_:MeOH, 9:1); UV-Vis (CHCl_3_): λ_max_ (log ε) = 577 nm (4.66); IR (ATR): *ν* = 3350*w,* 1596*s*, 1195*s*, 1298*s*, 1197*s*, 1138*s* cm^−1^; ^1^H NMR (400 MHz, CDCl_3_): δ = 7.63–7.52 (*m*, 2H, 41-H, 44-H), 7.50–7.43 (*m*, 1H, 42-H), 7.24–7.20 (*m*, 1H, 43-H), 6.72–6.59 (*m*, 2H, 47-H), 4.42–4.33 (*m*, 1H, 3-H), 3.86–3.01 (*m*, 17H, 18-H, 32-H, 34-H, 35-H, 37H, 49-H, 51-H), 2.97–2.85 (*m*, 4H, 54-H), 2.76–2.54 (*m*, 4H, 49-H), 2.52–2.43 (*m*, 1H, 13-H), 2.18–2.06 (*m*, 4H, 21-H_b_, 29-H), 2.07–1.99 (*m*, 4H, 55-H), 1.96 (*s*, 4H, 19-H, 31-H), 1.94–1.64 (*m*, 7H, 50-H, 22-H_a_, 16-H_a_, 15-H_a_), 1.60–1.14 (*m*, 18H, 1-H_a_, 2-H, 21-H_b_, 22-H_b_, 6-Ha, 16-H_b_, 11-H_a_, 7-H, 6-H_b_, 11-H_b_, 15-H_b_), 1.08–0.96 (*m*, 3H, 1-H_b_, 12-H), 0.90 (*s*, 3H, 24-H), 0.85 (*s*, 3H, 25-H), 0.80 (*s*, 3H, 27-H), 0.76 (*s*, 3H, 23-H), 0.71 (s, 3H, 26-H), 0.69–0.65 (m, 1H, 5-H) ppm; ^13^C NMR (101 MHz, CDCl_3_): δ = 213.2 (C-20), 170.9 (C-28, C-30, C-38), 151.9 (C-52), 151.2 (C-45), 136.6 (C-57), 136.3 (C-39, C-40), 130.4 (C-43), 129.4 (C-41, C-44), 126.8 (C-42), 126.7 (C-47), 123.5 (C-46), 113.0 (C-48), 105.2 (C-53), 80.8 (C-3), 55.4 (C-5), 53.0 (C-19), 50.9 (C-32, C-34, C-35, C-37), 50.6 (C-9), 50.4 (C-51, C-56), 50.3 (C-18), 49.8 (C-17), 44.1 (C-13), 41.8 (C-14), 40.6 (C-8), 38.3 (C-1), 37.7 (C-10), 37.1 (C-4), 35.8 (C-22), 34.2 (C-7), 31.8 (C-21), 30.1 (C-29), 29.9 (C-15), 28.8 (C-16), 27.9 (C-23), 27.5 (C-49), 27.3 (C-12), 23.6 (C-2), 22.6 (C-33, C-36), 21.2 (C-31), 21.1 (C-11), 20.6 (C-50), 19.9 (C-54), 19.6 (C-55), 18.1 (C-6), 16.4 (C-26), 15.9 (C-25), 14.7 (C-24), 14.0 (C-27) ppm; MS (ESI, MeOH/CHCl_3_, 4:1): *m*/*z* = 1070 (100%, [M-Cl]^+^); analysis calcd. for C_69_H_89_N_4_O_6_Cl (1105.94): C 74.94, H 8.11, N 5.07; found: C 74.73, H 8.35, N 4.81.

### 4.23. (2α,3ß,4α)2,3,23-Tris(acetoxy)-28-[4-[3-(2,3,6,7,12,13,16,17-octahydro-1H,5H,11H,15H-pyrido[3,2,1-ij]pyrido[1’’,2’’,3’’:1’,8’]quinolino[6’,5’:5,6]pyrano[2,3-f]quinolin-4-ium-9-yl)benzoyl]1,5-diazocan-1-yl]-28-oxo-olean-12-en Chloride (**28**)

Following GP 3 from **18** (200 mg, 0.3 mmol) and rhodamine 101 (200 mg, 0.4 mmol), followed by chromatography (silica gel, CHCl_3_/MeOH, 9:1), **28** (232 mg, 64%) was obtained as a pink solid; m.p. = 193–196 °C; R_f_ = 0.45 (CHCl_3_:MeOH, 9:1); UV-Vis (CHCl_3_): λ_max_ (log ε) = 578 nm (4.50); IR (ATR): *ν* = 2924*w,* 1739*w*, 1594*s*, 1493*s*, 1459*m*, 1361*m*, 1293*s*, 1195*s*, 1180*s*, 1090*s*, 1035*s*, 729*m,* 622*m*, 421*s* cm^−1^; ^1^H NMR (400 MHz, CDCl_3_): δ =7.67–7.58 (*m*, 2H, 47-H, 49-H), 7.52–7.48 (*m*, 1H, 46-H), 7.29–7.27 (*m*, 1H, 48-H), 6.77–6.65 (*m*, 2H, 52-H), 5.17–5.03 (*m*, 3H, 12-H, 2-H, 3-H), 3.83–3.79 (*m*, 1H, 23-H_a_), 3.60–3.16 (*m*, 17H, 23-H_b_, 37-H, 39-H, 40-H, 42-H, 56-H, 61-H), 3.00–2.94 (*m*, 4H, 59-H), 2.77–2.63 (*m*, 4H, 54-H), 2.46–2.36 (*m*, 1H, 18-H), 2.07 (*s*, 4H, 60-H), 2.06 (*s*, 3H, 36-H), 2.04–2.01 (*m*, 1H, 1-H_a_), 1.99 (*s*, 3H, 34-H), 1.95 (*s*, 7H, 32-H, 55-H), 1.91–1.86 (*m*, 2H, 11-H), 1.60–1.57 (*m*, 1H, 9-H), 1.46–1.42 (*m*, 2H, 21-H_a_, 22-H_b_), 1.35–1.30 (*m*, 4H, 6-H, 19-H, 5-H), 1.26–1.22 (*m*, 12H, 16-H_a_, 38-H, 41-H, 22-H_b_, 7-H, 15-H, 21-H_b_, 16-H_b_), 1.11–1.09 (*m*, 1H, 1-H_b_), 1.04 (*s*, 3H, 24-H), 0.98–0.94 (*m*, 1H, 20-H), 0.91 (*s*, 3H, 29-H), 0.85 (*s*, 3H, 25-H), 0.83 (*s*, 3H, 27-H), 0.82 (*s*, 3H, 30-H), 0.72 (*s*, 3H, 26-H) ppm; ^13^C NMR (101 MHz, CDCl_3_): δ = 176.3 (C-28), 170.8 (C-35, C-43), 170.4 (C-33), 170.3 (C-31), 152.0 (C-57), 151.3 (C-50), 139.1 (C-62), 136.6 (C-44), 130.3 (C-45), 129.6 (C-48), 129.2 (C-46) 129.1 (C-49), 127.0 (C-47, C-52), 124.5 (C-12), 123.4 (C-51), 113.0 (C-53), 105.2 (C-58), 74.9 (C-3), 69.9 (C-2), 65.3 (C-23), 55.6 (C-18), 51.0 (C-34, C-37, C-40, C-42), 50.5 (C-56, C-61), 47.7 (C-5), 47.5 (C-9), 46.2 (C-17), 43.7 (C-1), 41.9 (C-4, C-14), 39.5 (C-19), 38.6 (C-20), 37.8 (C-10), 32.6 (C-22), 31.9 (C-7), 30.6 (C-21), 29.7 (C-15), 29.6 (C-16), 27.6 (C-54), 23.3 (C-11), 22.6 (C-38, C-41), 22.6 (C-27), 21.2 (C-29), 21.0 (C-32), 20.8 (C-36), 20.7 (C-34), 20.6 (C-55), 19.9 (C-59), 19.7 (C-60), 17.9 (C-6), 17.4 (C-30), 17.1 (C-24), 17.0 (C-26), 14.1 (C-25) ppm; MS (ESI, MeOH/CHCl_3_, 4:1): *m*/*z* = 1084.3 (100%, [M-Cl]^+^); analysis calcd. for C_74_H_95_N_4_O_9_Cl (1220.04): C 72.85, H 7.85, N 4.59; found: C 72.63, H 8.01, N 4.39.

### 4.24. Cell Culture

Breast cancer cell lines were obtained from the Department of Radiobiology (MLU Halle-Wittenberg) and previously described. MDA-MB-231, HS578T and MCF-7, and T47D were cultured as a monolayer in RPMI (Thermo Fisher Scientific, Waltham, MA, USA) with 10% fetal bovine serum (Capricorn Scientific, Ebsdorfergrund, Germany), 2% penicillin/streptomycin (Sigma-Aldrich, St. Louis, MO, USA), and 1% sodium pyruvate (Gibco, Thermo Fisher Scientific) at 37 °C and 5% CO_2_. All cell lines were regularly tested for mycoplasma contamination.

### 4.25. SRB Assay

Breast cancer cells were seeded in 96 well plates with different cell numbers depending on the cell line in triplicate and after 24 h treated with different concentrations of compounds **14**–**28**. Treatment ended after 96 h when cells were fixed with 10% trichloroacetic acid (Carl Roth GmbH, Karlsruhe, Germany) for 1h at 4 °C. Afterwards, cells were washed with ice water four times and stained with 4.4% SRB solution (Sigma–Aldrich) for 10 min at room temperature. After washing cells with 1% acetic acid (Carl Roth GmbH), cells were air-dried overnight and then dissolved with 300 µL 20 mM Tris base solution (Sigma-Aldrich). Excitation was measured at 540 nm with a Spark plate reader (Tecan Treading AG, Männedorf, Switzerland) and IC_50_ values were calculated by dose-response curve fitting using Origin 2019 (OriginLab Corp., Northampton, MA, USA).

### 4.26. Cell Death

For the determination of apoptotic and necrotic cell death after treatment with compound 28, Annexin V-Sytox Deep Red staining was performed. Therefore, MDA-MB-231 and HS578T cells were seeded in 6-well plates. After 24 h, the cells were treated with different concentrations of compound 28 (10 nM, 100 nM, 250 nM, 500 nM, 1 µM, and 2 µM) for 24 h, 48 h, and 72 h at 37 °C and 5% CO_2_. For analysis of cell death, detached cells were collected in tubes and living cells were detached by accutase (Biowest, Nuaillé, France) and collected in the same tube. After several washing steps cells were resuspended in 1x annexin V binding puffer (10 mM HEPES, 140 mM NaCl, 2.5 mM CaCl_2_) and stained with 5 µL Annexin V-FITC (BioLegend, San Diego, CA, USA) and 1 µL 100 µM Sytox Deep Red Nucleic Acid Stain (Invitrogen, Thermo Fisher Scientific) for 15 min. Afterward, 400 µL 1x annexin V binding puffer were added to each tube. Gating was realized by the use of unstained, single annexin V-FITC or single Sytox Deep Red Nucleic Acid-stained cells, respectively. For quantification of necrotic and apoptotic cells, 10,000 cells were analyzed by LSRFortessa™ flow cytometer (BD Biosciences, Heidelberg, Germany).

### 4.27. Proliferation

MDA-MB-231 and HS578T cells were seeded in 6-well plates and treated with different concentrations (10 nM, 100 nM, 250 nM, 500 nM, 1 µM, and 2 µM) of compound 28 after 24 h. The number of dead and viable cells was measured by use of a CASY cell counter (OMNI Life Science, Bremen, Germany) after 72 h.

### 4.28. Staining

Analysis of subcellular localization of compound AS101 was performed in MDA-MB-231 cells using the mitochondrial targeting compound BioTracker™ 488 Green Mitochondria Dye (Sigma-Aldrich Chemie GmbH, Taufkirchen, Germany) for comparison. Cells were seeded in a µ-Plate 96 Well Black plate (ibiTreat: #1.5 polymer coverslip bottom, ibidi GmbH, Gräfelfing, Germany) at a cell density of 50,000 per well. After 24 h, cells were treated with 100 nM AS101 for 6h or 100 nM BioTracker488 for 30 min, followed by rinsing and supplementation with RPMI 1640 w/o Phenol-red (Pan-Biotech GmbH, Aidenbach, Germany). Live-cell imaging was performed on an Axio Observer 7 (Carl Zeiss Microscopy Deutschland GmbH, Oberkochen, Germany) using the settings for Ex/Em as follows: BioTracker (475 nm/514 nm), AS101 (555 nm/592); Scale bar: 50 µm.

## Figures and Tables

**Table 1:** Cytotoxicity of compounds **14**–**28** determined by SRB assay in four different breast cancer cell lines (MDA-MB-231, HS578T, MCF-7, and T47D). IC_50_ values were calculated after 96 h treatment. The data represent values of at least three independent experiments, which were done each in triplicate.

Compound	MDA-MB-231	HS578T	MCF-7	T47D
14 (µM)	2.88 ± 0.11	3.39 ± 0.92	3.03 ± 0.22	3.86 ± 0.93
15 (µM)	38.91 ± 14.08	15.18 ± 7.18	26.09 ± 10.76	49.67 ± 13.92
16 (µM)	3.36 ± 0.22	4.14 ± 0.13	3.59 ± 0.21	4.39 ± 0.88
17 (µM)	2.58 ± 0.37	2.77 ± 0.41	2.82 ± 0.57	3.78 ± 0.74
18 (µM)	0.46 ± 0.21	2.80 ± 0.16	1.53 ± 0.23	1.97 ± 0.29
19 (nM)	35.87 ± 19.42	280.06 ± 31.25	147.26 ± 68.02	190.96 ± 113.70
20 (nM)	71.76 ± 46.35	215.54 ± 96.53	155.25 ± 64.67	269.61 ± 76.07
21 (nM)	126.46 ± 40.55	351.94 ± 127.31	221.96 ± 90.61	261.83 ± 49.91
22 (nM)	134.05 ± 76.38	356.46 ± 92.90	120.63 ± 43.11	187.07 ± 60.55
23 (nM)	55.99 ± 19.44	275.88 ± 64.62	25.97 ± 21.28	32.71 ± 24.35
24 (nM)	1140.71 ± 255.22	1341.56 ± 74.91	1189.47 ± 325.25	1316.63 ± 713.38
25 (nM)	69.68 ± 8.43	341.79 ± 36.15	138.65 ± 111.56	232.17 ± 65.43
26 (nM)	135.93 ± 71.83	538.92 ± 27.80	239.90 ± 3.63	251.17 ± 56.18
27 (nM)	62.91 ± 22.03	440.34 ± 206.56	103.85 ± 19.75	129.25 ± 38.29
28 (nM)	0.60 ± 0.11	125.79 ± 7.61	3.96 ± 1.95	8.18 ± 6.51

## Data Availability

The data presented in this study are available on request from the corresponding author.
